# Angiogenic Imbalance and Inflammatory Biomarkers in the Prediction of Hypertension as Well as Obstetric and Perinatal Complications in Women with Gestational Diabetes Mellitus

**DOI:** 10.3390/jcm11061514

**Published:** 2022-03-10

**Authors:** Almudena Lara-Barea, Begoña Sánchez-Lechuga, Antonio Campos-Caro, Juan Antonio Córdoba-Doña, Raquel de la Varga-Martínez, Ana I. Arroba, Fernando Bugatto, Manuel Aguilar-Diosdado, Cristina López-Tinoco

**Affiliations:** 1 Department of Endocrinology and Nutrition, Puerta del Mar Hospital, 11009 Cádiz, Spain; almlarbar@gmail.com (A.L.-B.); bsanchezle@gmail.com (B.S.-L.); anaarroba@gmail.com (A.I.A.); manuelaguilardiosdado@gmail.com (M.A.-D.); 2 Biomedical Research and Innovation Institute of Cádiz (INiBICA), Puerta del Mar Hospital, 11009 Cádiz, Spain; antonio.campos@uca.es (A.C.-C.); jantonio.cordoba.sspa@juntadeandalucia.es (J.A.C.-D.); raqueldelavarga@hotmail.com (R.d.l.V.-M.); fgbugatto@yahoo.com (F.B.); 3 Department of Biomedicine, Biotechnology and Public Health, Genetic Area, Cadiz University (UCA), 11003 Cádiz, Spain; 4 Preventive Medicine and Public Health Unit, Jerez University Hospital, 11407 Jerez de la Frontera, Spain; 5 Department of Immunology, Puerta del Mar Hospital, 11009 Cádiz, Spain; 6 Department of Obstetrics and Gynaecology, Puerta del Mar Hospital, 11009 Cádiz, Spain; 7 Area of Obstetrics and Gynaecology, Department of Child and Mother Health and Radiology, Medical School, Cadiz University (UCA), 11003 Cádiz, Spain; 8 Department of Medicine, Cadiz University (UCA), 11003 Cádiz, Spain

**Keywords:** gestational diabetes, hypertensive disorders of pregnancy, angiogenic factors, sFlt-1/PIGF ratio

## Abstract

Gestational diabetes mellitus (GDM) increases the risk of hypertensive disorders of pregnancy (HDP). We aimed to analyze the altered inflammatory markers and angiogenic factors among women with GDM to identify pregnant women at higher risk of developing HDP. Methods: This was a prospective study of 149 women without hypertension diagnosed in the third trimester with GDM. Inflammatory markers and angiogenic factors were measured at 28–32 weeks of pregnancy. Obstetric and perinatal outcomes were evaluated. Results: More than eight percent of the women developed HDP. Higher levels of the soluble fms-like tyrosine kinase-1/placental growth factor (sFlt-1/PIGF) ratio (4.9 ± 2.6 versus 2.3 ± 1.3, respectively; *p* < 0.001) and leptin (10.9 ± 0.8 versus 10.08 ± 1.1, respectively; *p* = 0.038), as well as lower levels of adiponectin (10.5 ± 1.3 versus 12.9 ± 2.7, respectively; *p* = 0.031), were seen in women who developed HDP versus normotensive women with GDM. A multivariable logistic regression analysis showed that adiponectin had a protective effect with 0.45-fold odds (0.23–0.83; *p* = 0.012), and that the sFlt-1/PIGF ratio was associated with 2.70-fold odds of developing HDP (CI 95%: 1.24–5.86; *p* = 0.012). Conclusion: An increase in angiogenic imbalance in the sFlt-1/PIGF ratio in women with GDM was detected and may be an indicator of developing HDP in addition to any subsequent obstetric and perinatal complications.

## 1. Introduction

Hypertensive disorders of pregnancy (HDP) are present in 5–10% of gestations worldwide and contribute to an increase in maternal and neonatal complications [1]. In pregnant women with pregestational diabetes, the risk is around 20%, compared to women without diabetes where it is around 5% [2]. Pregnant women with gestational diabetes mellitus (GDM) also have an increased risk of HDP compared with non-GDM women [3], although the rate is variable due to the low disease prevalence of both conditions, the lack of a universal disease classification, and interdependent risk factors for both GDM and HDP [4].

One hypothesis claims that the association between GDM and preeclampsia could be—at least partly—due to insulin resistance and its adaptation in normal pregnancy, whereas in individuals predisposed to other risk factors this could lead to pathological processes, such as the development of GDM and preeclampsia [5,6].

Circulating adipokines have been involved in the pathophysiology of insulin resistance; therefore, they could be potential candidate biomarkers for preeclampsia. Prospective studies illustrate the link between the downregulation of adiponectin as well as anti-inflammatory cytokines (e.g., IL-4 and IL-10) and the upregulation of leptin as well as proinflammatory cytokines implicated in insulin resistance (e.g., IL-6 and TNF-α) [7,8].

The role of pro- and anti-inflammatory markers as biomarkers for developing preeclampsia in type 1 diabetes mellitus has been demonstrated in several studies [9]. A recent study on pregnant women with type 1 diabetes mellitus has shown that, as early as the first trimester, the profiles of adipokine biomarkers related to insulin resistance (adiponectin, resistin, and others) increase the incidence of preeclampsia [2]. It has been observed that antiangiogenic-soluble fms-like tyrosine kinase-1 (sFlt-1) levels are elevated before the development of preeclampsia (5 weeks before) [10], whereas levels of the angiogenic placental growth factor (PIGF) have been shown to diminish in women with preeclampsia [11]. Other biomarkers, such as plasminogen activator inhibitor-1 (PAI-1), have also been involved previously in the pathogenesis of preeclampsia, due to a key role in the regulation of local inflammatory processes [12]. However, few studies have evaluated these inflammatory markers and angiogenic factors during pregnancy in women with GDM, and, to our knowledge, there are no studies that have analyzed their relationship to obstetric and perinatal complications. It is likely that hyperglycemia, insulin resistance, as well as cytokine release during hyperglycemia may play a crucial role in the pathogenesis of endothelial dysfunction, which leads to the development of HDP.

We aim to study the presence of inflammatory markers and angiogenic factors in the screening of women with GDM and their potential role as predictive factors for identifying pregnant women at an elevated risk of developing HDP and obstetric and perinatal complications. This research may help to demonstrate the pathogenic mechanisms for gestational hypertension, preeclampsia, and their long-term sequelae, as well as assess the role of inflammatory factors in the screening and development of preventive and therapeutic strategies for HDP in GDM.

## 2. Materials and Methods

### 2.1. Study Design and Study Population

The study was accepted by the Hospital Research Ethics Board (Puerta del Mar Hospital) according to the principles of the Declaration of Helsinki. This is a prospective cohort study conducted from 2014 to 2018 on women attending the High-Risk Pregnancies clinic at Puerta del Mar University Hospital, Cadiz, Spain. One hundred and sixty-nine normotensive pregnant women with a diagnosis of GDM were selected between 28 and 32 weeks of gestation. Ninety-four healthy pregnant women were invited to volunteer and participate as the control group. They signed specially designed informed consent forms to participate in the study and then were followed throughout their pregnancy. The inclusion criteria were the absence of prepregnancy diabetes, hypertension, morbid obesity, concomitant chronic or acute systemic disease, placental insufficiency, and an active tobacco habit.

Diagnosis of GDM was established in the 2nd or 3rd trimester of gestation using a two-step approach according to the criteria of the National Diabetes Data Group [13]: in all pregnant women between 24 and 28 weeks of gestation (and women with risk factors during the first trimester of pregnancy) a screening test was performed with a 50 g glucose test. Women with a positive screening test (1-h blood glucose > 140 mg/dl (7.7 mmol/L) underwent a confirmatory 3-h, 100 g oral glucose tolerance test. GDM was diagnosed with two abnormally high values of the following thresholds; fasting glucose, 105 mg/dL (5.8 mmol/L); 1-h, 190 mg/dL (10.5 mmol/L); 2-h, 165 mg/dL (9.1 mmol/L); 3-h, 145 mg/dL (8.0 mmol). All women with GDM received complex dietary counselling at the diagnosis time, and they were educated to monitor their blood glucose levels. The target glucose level in the treatment of GDM was a fasting blood glucose of less than 5.3 mmol/L, and less than 7.8 mmol/L for 1 h after a meal. Those who failed to achieve target glucose levels were treated with insulin.

### 2.2. Evaluation of Variables Collected

Clinical data and specimen blood samples were collected at 28–32 weeks. Two measurements of blood pressure (BP) were taken with an automated BP monitor Omron HEM-7200-E (Kyoto, Japan) to rule out chronic hypertension. At the end of the pregnancy we considered the presence of HDP, including gestational hypertension and preeclampsia. Gestational hypertension was defined as hypertension (>140/90 mmHg) in a woman who was normotensive before the 20th week of gestation and whose BP returned to normal by 12 weeks after delivery. Preeclampsia was defined as the new onset of hypertension after the 20th week of gestation in a previously normotensive woman, and develops proteinuria or end-organ dysfunction [14].

The following obstetric and perinatal data were consulted after delivery: gestational age at delivery, route of delivery, birthweight, and the Apgar score of the newborn. Preterm delivery was considered as a delivery that occurred before 37 gestational weeks. Small for gestational age (SGA) was defined as a birthweight below the 10th percentile. Macrosomia and large for gestational age (LGA) were designated as a birthweight above the 95th and 90th percentile for gestational age, respectively.

### 2.3. Laboratory Measurements

Venous blood samples for biochemical analysis were obtained in the morning, after an overnight fast, from an antecubital vein between 8.00 AM and 9.00 AM through the use of vacuum tubes. Samples were centrifuged at 3000× *g* for 7 min, and aliquots were obtained and stored at −80 °C in a freezer before use. Assays were performed by personnel who were unaware of the outcome of the pregnancy. We measured sFlt-1, PIGF, adiponectin, leptin, MCP1, PAI-1, resistin, NGF, TNFα, HGF, and FGF2. All cytokine levels were measured in plasma using commercial kits according to the manufacturer′s recommendations (Millipore, Billerica, MA, USA) through xMAP technology (Luminex Corporation, Austin, TX, USA). Concentrations of cytokines were reported as picograms per milliliter. A logarithmic scale was used with cytokines due to large values.

### 2.4. Statistical Analysis

We present the descriptive statistics of the variables as the mean, median, standard deviation (SD), or percentages for quantitative or qualitative variables. We used the Shapiro–Wilk test to monitor the normality of the distributions. Comparisons between quantitative variables and groups were carried out with the Student′s *t*–test and one-way analysis of variance (ANOVA) for the parametric variables. To identify significant differences between specific groups, Bonferroni post hoc tests were used for equal variances and Games–Howell post hoc tests were used in cases of unequal variances. The Mann–Whitney *U* test and Kruskal–Wallis *H* test were used for nonparametric variables. Correlations were evaluated with Pearson’s correlation for parametric variables and Spearman’s test for nonparametric variables. A multivariate analysis was performed using nonconditional logistic regression, considering potential factors associated with insulin resistance, such as body mass index (BMI) and age. The stepwise technique was used to select the independent variables introduced into the model. Receiver operating characteristics (ROC) curves were developed to evaluate the ability of each index to predict HDP states. Statistical significance was established at *p* ≤ 0.05.

## 3. Results

**Figure 1:**
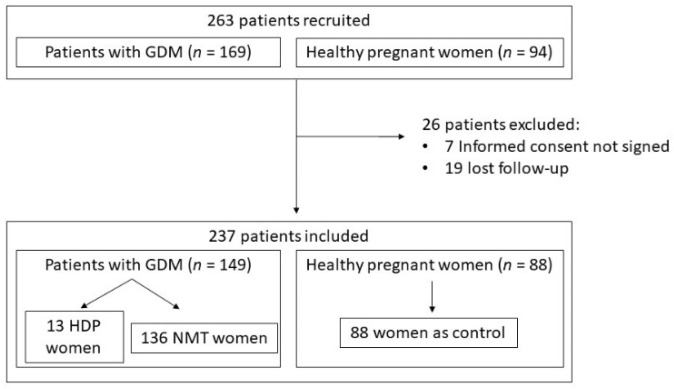
Study flow chart: algorithm for the identification of eligible women and number of women for each step of the procedure. GDM: gestational diabetes mellitus; HDP: hypertensive disorders of pregnancy; NMT: normotensive.

Two hundred and thirty-seven patients completed the study: 149 pregnant women with GDM and 88 controls. Thirteen women with GDM (8.7%) developed HDP, nine presented with gestational hypertension (6%), and four developed preeclampsia (2.7%). None of the pregnant women in the control group developed HDP (Figure 1). We retrospectively analyzed the demographic, clinical, and laboratory variables at the time of inclusion between women who developed HDP versus the normotensive GDM group and control group (Table 1). We found that diastolic BP was significantly higher in women who developed HDP compared with the non-HDP groups, including diabetic and nondiabetic women, when blood pressure was measured at 28–32 weeks of pregnancy. Pregestational BMI and levels of HbA1c were also significantly higher in women with HDP than in the other groups. The other variables did not show significant differences.

Concerning the obstetric and perinatal outcomes, the gestational age at delivery, birthweight, and customized percentile were significantly lower in women with HDP compared to the normotensive GDM group and control group, as shown in Table 2. We also found a significantly higher rate of SGA and intrauterine growth restriction (IUGR) in HDP women. No differences in the rest of the studied variables were detected.

Among women with GDM, eighty-three (55.7%) of them were treated with a diet and sixty-six (44.3%) received insulin. Women in the insulin group had a greater proportion of a family history of diabetes mellitus (59.1 vs. 39.8%, respectively, *p* = 0.019) and a personal history of GDM (31.8 vs. 14.5%, respectively, *p* = 0.011). They also had significantly higher mean fasting glucose levels (5.36 ± 0.64 vs. 4.78 ± 0.49 mmol/L, respectively, *p* < 0.001), HbA1c levels (5.11 ± 0.47 vs. 4.96 ± 0.32%, respectively, *p* = 0.02), and HOMA index (2.45 ± 1.83 vs. 1.31 ± 0.86, respectively *p* = 0.035) compared with the diet group. There were no significant differences in HDP development and obstetrical or perinatal complications between the group treated with insulin and that treated with a diet.

Data on cytokines’ and angiogenic factors’ levels are shown in Table 3. Women with HDP presented higher concentrations of leptin and MCP-1, and sFlt-1/PIGF ratio in addition to lower adiponectin and PlGF levels compared to the normotensive groups. We did not observe significant differences in the rest of the markers between the groups. Correlation analyses showed a significant positive association between leptin and BP at the time of inclusion (r = 0.24, *p* = 0.002). No significant correlation was found between systolic BP and the rest of the cytokines. For diastolic BP at the time of inclusion, there was a significant negative correlation with PlGF (r = −0.36, *p* < 0.001), and positive associations between MCP-1 (r = 0.17, *p* = 0.04), leptin (r = 0.28, *p* < 0.001), and sFlt-1/PIGF levels (r = 0.38, *p* < 0.001) were found.

**Figure 2:**
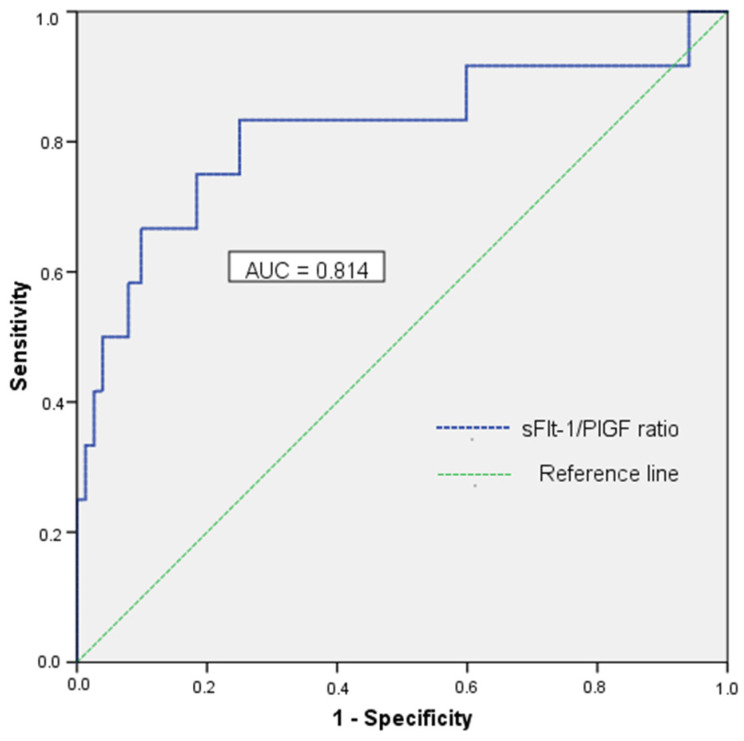
Receiver operating characteristic curve for the sFlt1/PlGF ratio. The ROC curve showing the sFlt1/PIGF ratio as the best predictor for HDP, with an AUC of 0.814.

In our study, the best predictor for HDP in women with GDM was the sFlt-1/PIGF ratio, with an AUC of 0.814 (CI 95%: 0.66–0.98) at a concentration threshold of 3.0 pg/mL, with a sensitivity of 83.3%, a specificity of 75%, an NPV of 96.8%, a positive likelihood value (LR+) of 3.33, and a negative likelihood ratio value (LR-) of 0.22 (Figure 2).

**Figure 3:**
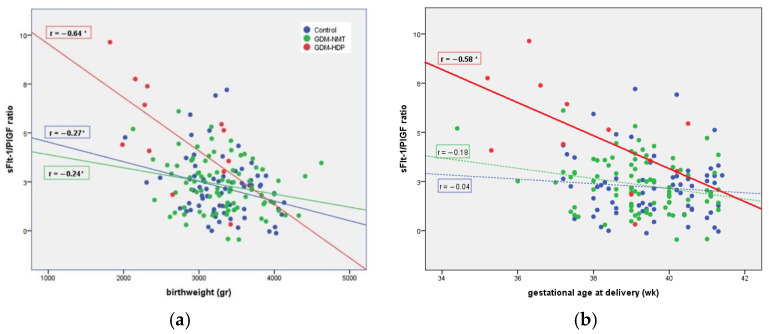
The correlations of plasma sFlt-1/PIGF ratio according to the birthweight (**a**) and the gestational age at delivery (**b**). (**a**) Correlation between the sFlt-1/PIGF ratio and birthweight. * *p* < 0.05. (**b**) Correlation between the sFlt-1/PIGF ratio and gestational age at delivery. * *p* < 0.05.

Correlation analyses showed significant negative associations between the sFlt-1/PIGF ratio and birthweight within the groups, with the correlation being strongest in the GDM-HDP group as shown in Figure 3a. For the gestational age at delivery, this correlation was statically significant only in the GDM-HDP group (Figure 3b). Furthermore, among women who subsequently developed HDP, we found maternal PAI-1 levels (11.11 ± 3.40 vs. 5.61 ± 2.95 pg/mL, respectively *p* = 0.013) and resistin levels (10.27 ± 3.31 vs. 5.09 ± 2.65 pg/mL, respectively, *p* = 0.012) significantly higher in the serum of patients with a pregnancy complicated by IUGR than in pregnancies without IUGR. We did not observe significant differences in the rest of the markers related to other perinatal complications.

Table 4 shows the final model of the multivariate analysis; the outcomes indicate that a high sFlt-1/PIGF ratio (OR: 2.70) increases the chance of developing HDP.

## 4. Discussion

Maternal insulin resistance has been implicated in the pathogenesis of HDP due to the existence of marked hyperinsulinemia during pregnancy before the development of HDP [5,6]. We consider it necessary to establish biomarkers during pregnancy in normotensive women with actual GDM to predict HDP and avoid maternal as well as neonatal complications associated with both diseases. This paper is the first prospective cohort study that seeks to assess the proinflammatory state of women with GDM and how it influences the further development of HDP and subsequent obstetric and perinatal complications. A proinflammatory cytokine pattern and, in particular, an increase in the sFlt-1/PIGF ratio in pregnant women with GDM were demonstrated, for the first time, to predict the development of HDP, an earlier gestational age at delivery, and a lower birthweight.

While the percentage of women who developed HDP was low in our study, the prevalence of HDP in women with GDM in the literature is unclear [3,5] due to low disease prevalence, the lack of a universal disease classification, differences among various population groups, as well as interdependent risk factors for both diseases [15]. Kvetny et al. [16] concluded that gestational hypertension was found more frequently in women with GDM (28%) than in women with normal glucose tolerance (10%). The Fifth International Workshop Conference [17] on GDM and the work of Roberts et al. [18] proved that GDM increased the risk of developing preeclampsia by two to three times; meanwhile, other authors [4] have described a lower and more variable prevalence in GDM. However, we can explain our low prevalence based on having selected only women with late-onset GDM for our study, avoiding those women who might present unrecognized type 1 or 2 diabetes prior to pregnancy that show a higher risk of cardiovascular diseases, including hypertension. Secondly, based on international guidelines [14,19], our hospital protocol includes low-dose aspirin prophylaxis for pregnant women with risk factors for HDP development, which accounts for a lower risk of developing HDP.

We found no differences in terms of the risk of developing HDP among pregnant women treated with a diet and those treated with insulin, as has been reported by some authors [20,21], and a recent meta-analysis of 10 trials (with three including preeclampsia as a secondary target) also found no significant association between insulin-treated GDM and preeclampsia [22]. However, we found that women undergoing insulin therapy had a longer history of previous GDM and a longer family history of diabetes mellitus, which is likely to be related to cumulative damage with each additional pregnancy and the ensuing decrease in the β-cell reserve [23], which would increase not only the risk of re-developing GDM but also the need for insulin therapy to maintain glycemic control. In contrast to other adipokines, plasma adiponectin correlates inversely with insulin resistance and diminishes with an increase in the insulin resistance as pregnancy advances [2,24], and, as previous studies described [8,25], lower adiponectin concentrations were detected in women with GDM compared to non-GDM women. An assessment of adiponectin in the prediction of HDP has shown contradictory results; while some authors found circulating adiponectin levels were elevated in women with preeclampsia [26,27], others, such as ourselves, demonstrated lower levels of adiponectin in women who develop subsequent gestational hypertension and preeclampsia [25,28]. Moreover, our finding of lower adiponectin levels in women with GDM and HDP compared to normotensive women with GDM further underscores the influence of insulin resistance on the development of HDP, suggesting adiponectin levels could be used as a potential biomarker to predict the development of HDP in women with GDM.

Regarding other proinflammatory cytokines, we agree with the studies that support higher maternal leptin levels in women who develop HDP [29,30,31]. As described in the literature, serum leptin concentrations are directly proportional to fat mass and are increased in obesity and pregnancy due to maternal weight gain as well as the placenta’s secretion, contributing to the insulin resistance and GDM pathophysiology [7]. In fact, several works have demonstrated higher leptin levels in women with GDM compared to healthy pregnant women [4,8,24,32]; however, this is the first study, to our knowledge, that has been able to detect, among women with GDM, higher levels of leptin in those that develop HDP compared to normotensive women with GDM. With respect to the influence of BMI/adiposity on leptin levels, conflicting results has been reported. Hendler et al. [29] found increased leptin levels in women with severe preeclampsia and overweight, but not in women of normal weight. In our cohort, although women with HDP had a greater BMI, HDP was associated with higher maternal leptin levels in both normal weight and obese women, in agreement with others publications [30,31]. In addition, we found greater levels of MCP-1 in diabetic women and even higher levels in diabetic women who developed HDP, suggesting a role in the activation of chronic inflammation in GDM [33,34]. As others have found, we were not able to detect many other proinflammatory cytokines associated with preeclampsia, such as resistin [25] or TNFα [2].

Related to the mechanisms potentially involved in our findings, Mordwinkin et al. [35] demonstrated in women with GDM a decrease in maternal circulating endothelial progenitor cells and an increase in soluble adhesion molecules together with a decrease in the expression of superoxide dismutase and an increase in endothelial nitric oxide synthase (NOS) expression. Furthermore, it seems that an altered angiogenic balance plays an important role in the pathogenesis of preeclampsia [10]. Our results show lower PIGF values in pregnant women with HDP, as has been previously reported in the literature [11,36,37,38]. Considering extensive evidence on sFlt-1 and PIGF factors in preeclampsia pathogenesis, diagnosis, and prediction, Verlohren et al. [10] confirmed that a one-unit increase in the log of the sFlt-1/PIGF ratio may raise the risk of developing HDP by 2.5 times. Nikuei et al. [39] reported 90% diagnostic accuracy, 84.2% sensitivity, 85% specificity, 91.4% PPV, and 73.9% NPV of the sFlt-1/PIGF ratio as a predictive marker of preeclampsia in the second term of DMG-free pregnant women. Râdelescu et al. [11] identified 96.6% sensitivity, 46.2% diagnostic accuracy, 40% PPV, and 100% NPV. In our work, the ratio sensitivity and specificity are somewhat lower than those reported by other studies; however, we must bear in mind that earlier research was conducted on healthy GDM-free pregnant women, which may have had an influence on such differences.

Interestingly, we showed that there is a statistically significant correlation between diastolic blood pressure and the sFlt-1/PIGF ratio in women with GDM. In this context, controversial results have been shown previously. A study comparing laboratory parameters in women with preeclampsia found a significant correlation between serum sFlt-1/PIGF ratio levels and systolic (r = 0.35) as well as diastolic (r = 0.30) BP values [40]. Staff et al. [41] also showed that maternal concentrations of sFlt-1 increased through augmenting the systolic BP in the preeclampsia group (r = 0.4, *p* = 0.03). In contrast, Verlohren et al. [42] showed a moderate correlation between systolic BP only in the <34 weeks preeclampsia group with the sFlt-1/PIGF ratio, and another study could not find a statistical difference [43]. Nevertheless, these studies did not include diabetic women; therefore, this is the first time that a statistical difference has been shown between the sFlt-1/PIGF ratio and diastolic BP values in women with GDM.

In terms of perinatal outcomes, the hypothesis exists that preeclampsia and IUGR are linked etiologically but have different clinical manifestations [43,44,45]; the link among lower PlGF and higher sFlt-1 levels in IUGR newborns of mothers with preeclampsia has been previously described [40,41,45,46]. Our results revealed no differences in both of these biomarkers in pregnancies complicated by IUGR among women who developed HDP, but we did find that the sFlt-1/PIGF ratio was negatively correlated with the week of pregnancy at delivery and the weight of the newborn in women with GDM who developed subsequent HDP, both of which were consistent with those reported by other authors [47,48]. On the other hand, it has been documented that PAI-1 plays a key role in the regulation of inflammation [49], and demonstrated increased PAI-1 concentrations may contribute to inflammation and a hypercoagulable state in preeclamptic women [12], which could reflect an endothelial disturbance [44,50]. Despite our results revealing no differences in PAI-1 levels between HDP and normotensive GDM women, we have demonstrated a link between higher maternal PAI-1 levels and IUGR in women with GDM and HDP, similar to Sheppard et al. [51], who found significantly higher placental PAI-1 levels in IUGR pregnancies with or without preeclampsia. This finding may prove a possible common pathway of altered placental perfusion that IUGR and HDP could share, consistent with the hypothesis that considers IUGR a step on the way to preeclampsia [52]. Similar to PAI-1 levels, we also found higher resistin levels in mothers of newborns with IUGR among GDM-HDP women; unfortunately, these results have never been revealed previously, and conflicting results about circulating resistin are reported in preeclampsia [25,29].

The role of the sFlt-1/PIGF ratio has been documented previously in several studies conducted on type 1 and type 2 diabetes mellitus [53,54]; recently, Nuzzo et al. [37] have also identified an increased placental and maternal sFlt1/PlGF ratio in women with GDM who develop preeclampsia.

Our results confirm these findings and enable us to demonstrate the sFlt-1 /PIGF ratio as a valid predictor of the development of HDP in women with real GDM. Furthermore, to the best of our knowledge, this is the first report associating the role of sFlt-1/PIGF ratio in GDM with perinatal and obstetric complications.

One of the strengths of our study is its longitudinal design and inclusion of women with a late onset of GDM diagnosed in the second trimester according to NDDG [13]. However, this study has some potential limitations, the most important being the small number of women who develop HDP, which could lead to overfitting in the logistic regression model, especially if using numerous independent variables. To minimize this potential drawback, we only included two predictor and two adjustment variables in the final model.

## 5. Conclusions

On the basis of the results of the current study, we conclude that higher sFlt-1/PIGF ratio contributed to the increased incidence of HDP and obstetric as well as perinatal complications in women with GDM. These findings are important because they suggest that cytokines may offer an alternative assessment of HDP risk as well as obstetric and perinatal complications in women with GDM, but our results would need to be confirmed with a larger and more reliable sample. Improved prediction would bring immediate benefit to the planning of antenatal care and set a precedent for the implementation of future studies that require selecting women at high risk of developing HDP. Knowledge of these findings would permit the elucidation of the pathogenic bases of the process and support the establishment of predictive, therapeutic, and preventative strategies.

## Figures and Tables

**Table 1:** Demographic, clinical, and laboratory variables at the time of inclusion.

Variable *	GDM-HDP (*n* = 13)	GDM-NMT (*n* = 136)	Control (*n* = 88)	*p*-Value
Age (y)	33.9 ± 3.7	34.6 ± 4.3	33.1 ± 5.1	ns
Pregestational BMI (kg/m^2^)	30.1 ± 6.8	26.8 ± 4.9	24.5 ± 3.8	<0.001 ^a,c^
Systolic BP (mmHg)	117.9 ± 16.1	109.4 ± 14.1	109.4 ± 11.9	ns
Diastolic BP (mmHg)	73.2 ± 10.9	64.8 ± 8.6	63.4 ± 8.7	0.001 ^a,b^
Basal glucose (mmol/L)	4.98 ± 0.49	4.97 ± 0.62	4.65 ± 0.32	0.042 ^c^
HbA1c (%)	5.24 ± 0.5	5.0 ± 0.4	4.8 ± 0.3	<0.001 ^a,c^
HOMA-IR	2.4 ± 0.9	1.6 ± 1.3	1.9 ± 1.1	ns
Albumin/creatinine (mg/g)	30.2 ± 62.1	66.7 ± 7.5	4.9 ± 3.6	<0.001
Uric acid (mmol/L)	0.25 ± 0.07	0.23 ± 0.13	0.23 ± 0.23	ns
Total cholesterol (mmol/L)	6.41 ± 0.65	6.40 ± 1.29	6.48 ± 1.0	ns
LDL cholesterol (mmol/L)	3.66 ± 0.49	3.82 ± 1.39	3.51 ± 0.98	ns
HDL cholesterol (mmol/L)	1.99 ± 0.44	1.85 ± 0.45	1.99 ± 0.5	ns
Triglycerides (mmol/L)	2.36 ± 0.62	2.27 ± 0.9	2.05 ± 0.72	ns

GDM = gestational diabetes mellitus; HDP = hypertensive disorders of pregnancy; NMT = normotensive; BMI = body mass index; BP = blood pressure; HbA1c = glycated hemoglobin; LDL = low-density lipoprotein; HDL = high-density lipoprotein; ns = not statistically significant. * Data expressed as means ± standard deviation. ^a^ Significant differences between the GDM-HDP and control groups. ^b^ Significant differences between the GDP-HDP and GDM-NMT groups. ^c^ Significant differences between the GDM-NMT and control groups.

**Table 2:** Obstetric and perinatal outcomes in women with HDP and without HDP.

Variable	GDM-HDP (*n* = 13)	GDM-NMT (*n* = 136)	Control (*n* = 88)	*p*-Value
Systolic BP (mmHg) *	151.7 ± 29.9	120 ± 12.3	119.8 ± 11.4	<0.001 ^a,b^
Diastolic BP (mmHg) *	90.5 ± 16.6	71.5 ± 10.2	70.2 ± 7.5	<0.001 ^a,b^
Gestational age at delivery (wk) *	37.9 ± 1.7	38.9 ± 1.4	39.6 ± 1.3	<0.001 ^a,b,c^
Cesarean section ^†^	5 (38.5%)	43 (31.6%)	21 (2.9%)	ns
Birthweight (g) *	2701 ± 591	3256 ± 477	3323 ± 429	<0.001 ^a,b^
Customized percentile *	24.3 ± 25	48.7 ± 28.7	48.4 ± 28.5	0.017 ^a,b^
Apgar < 6 at 5 min ^†^	0	2 (1.5%)	0	ns
Macrosomia ^†^	0	17 (12.5%)	10 (11.4%)	ns
LGA ^†^	0	16 (11.8%)	8 (9.1%)	ns
SGA ^†^	6 (46.2%)	11 (8.1%)	11 (12.5%)	<0.001
IUGR ^†^	4 (30.8%)	5 (3.7%)	7 (8%)	0.001

GDM = gestational diabetes mellitus; HDP = hypertensive disorders of pregnancy; NMT = normotensive; BP = blood pressure; LGA = large for gestational age; SGA = small for gestational age; IUGR = intrauterine growth restriction; ns = not statistically significant. * Data expressed as means ± standard deviation. ^†^ Data expressed as *n* (%). ^a^ Significant differences between the GDM-HDP and control groups. ^b^ Significant differences between the GDP-HDP and GDM-NMT groups. ^c^ Significant differences between the GDM-NMT and control groups.

**Table 3:** Levels of soluble markers analyzed in women with HDP and without HDP.

Variable *	GDM-HDP (*n* = 13)	GDM-NMT (*n* = 136)	Control (*n* = 88)	*p*-Value
Adiponectin (pg/mL)	10.52 ± 1.33	12.94 ± 2.78	13.18 ± 2.97	0.031 ^a,b^
Resistin (pg/mL)	6.69 + 3.69	7.21 + 3	8.31 + 3.31	0.028 ^c^
PAI-1 (pg/mL)	7.31 ± 3.96	7.65 ± 3.15	8.79 ± 3.45	0.034 ^c^
NGF (pg/mL)	0.34 ± 0.75	0.63 ± 0.83	0.92 ± 0.92	ns
Leptin (pg/mL)	10.97 ± 0.85	10.08 ± 1.14	10.16 ± 0.99	0.038 ^b^
HGF (pg/mL)	6.51 ± 1.08	7.1 ± 1.01	6.87 ± 1.91	ns
MCP-1 (pg/mL)	5.23 ± 0.61	5.03 ± 0.53	4.81 ± 0.58	0.023
TNFα (pg/mL)	0.18 ± 2.05	0.51 ± 1.11	0.79 ± 0.75	ns
sFlt-1 (pg/mL)	7.58 ± 1.08	7.7 ± 0.95	7.41 ± 1.0	ns
PIGF (pg/mL)	2.66 ± 1.95	5.05 ± 1.05	5.32 ± 1.06	<0.001 ^a,b^
FGF2 (pg/mL)	3.79 ± 0.75	4.08 ± 0.63	4.06 ± 0.65	ns
sFlt-1/PlGF ratio	4.92 ± 2.63	2.34 ± 1.33	2.21 ± 1.56	<0.001 ^a,b^

GDM = gestational diabetes mellitus; HDP = hypertensive disorders of pregnancy; NMT = normotensive; PAI-1 = plasminogen activator inhibitor-1; NGF = nerve growth factor; HGF = hepatocyte growth factor; MCP-1 = monocyte chemoattractant protein-1; TNFα = tumor necrosis factor alpha; sFlt-1 = soluble fms-like tyrosine kinase-1; PIGF = placental growth factor; FGF2 = fibroblast growth factor 2; ns = not statistically significant. * Data expressed as means ± standard deviation. ^a^ Significant differences between the GDM-HDP and control groups. ^b^ Significant differences between the GDP-HDP and GDM-NMT groups. ^c^ Significant differences between the GDM-NMT and control groups.

**Table 4:** Multivariate logistic regression for HDP in women with GDM.

Variable	OR	Z-Score	*p*-Value	CI 95%
Age (y)	0.94	−0.05	0.76	(0.67–1.32)
Pregestational BMI (kg/m^2^)	1.14	0.13	0.32	(0.87–1.50)
Adiponectin (pg/mL)	0.45	−0.80	0.012	(0.23–0.83)
Leptin (pg/mL)	1.04	0.04	0.96	(0.18–5.86)
sFlt-1/PIGF ratio	2.70	0.99	0.012	(1.24–5.86)

OR: odds ratio; 95% CI: 95% confidence interval.

## Abbreviations

AUC	area under the curve
BMI	body mass index
BP	blood pressure
FGF2	fibroblast growth factor 2
GDM	gestational diabetes mellitus
HbA1c	glycated hemoglobin
HDL	high-density lipoprotein
HDP	hypertensive disorders of pregnancy
HGF	hepatocyte growth factor
IUGR	intrauterine growth restriction
LDL	low-density lipoprotein
LGA	large for gestational age
MCP-1	monocyte chemoattractant protein-1
NGF	nerve growth factor
NOS	nitric oxide synthase
NMT	normotensive
NPV	negative predictive value
ns	not statically significant
PAI-1	plasminogen activator inhibitor-1
PlGF	placental growth factor
PPV	positive predictive value
SD	standard deviation
sFlt-1	soluble fms-like tyrosine kinase-1
SGA	small for gestational age
TNFα	tumor necrosis factor alpha
